# Resting‐State Functional Connectivity of Sensorimotor and Default Mode Networks and Lower Limb Performance in Chronic Stroke: A Cross‐Sectional Study

**DOI:** 10.1002/brb3.70519

**Published:** 2025-05-07

**Authors:** Svetlana Pundik, Margaret M. Skelly, Jessica P. McCabe, Ahlam I. Salameh, Trenley Anderson, Kelsey Rose Duncan, Terri Hisel, Sarah J. A. Carr

**Affiliations:** ^1^ Department of Veterans Affairs Northeast Ohio Healthcare System (VANEOHS) Cleveland Ohio USA; ^2^ School of Medicine Case Western Reserve University Cleveland Ohio USA; ^3^ Department of Biomedical Engineering Case Western Reserve University Cleveland Ohio USA; ^4^ Division of Preclinical Sciences Kent State University College of Podiatric Medicine Independence Ohio USA; ^5^ University Hospitals of Cleveland Case Medical Center Cleveland Ohio USA; ^6^ School of Neuroscience, Institute of Psychiatry, Psychology and Neuroscience King's College London London UK

**Keywords:** Keywords, gait, lower extremity, neural networks, neuronal plasticity, resting‐state functional magnetic resonance imaging, stroke

## Abstract

**Introduction:**

Stroke disrupts functional brain connectivity, yet how this relates to lower limb motor and sensory abilities is not well understood. Greater knowledge of movement‐related brain connectivity can aide in the development of better interventions for recovery after stroke. Our objective was to evaluate the relationship between lower limb performance and resting‐state functional connectivity (rsFC) of large‐scale brain networks for individuals with chronic motor deficits (> 6 months) after stroke.

**Methods:**

Resting‐state functional magnetic resonance imaging and lower limb clinical measures were collected for 37 individuals. Regions of interest (ROI)‐to‐ROI connectivity analysis was conducted for cortical sensorimotor network (SMN) (cortical SMN), SMN with both cortical and subcortical ROIs (SMN), and default mode network (DMN). Relationship of ROI–ROI connectivity and clinical measures or lesion load was assessed using general linear models. Graph theory metrics (global efficiency, clustering coefficient, and betweenness centrality) were related to clinical measures using elastic nets analysis.

**Results:**

Greater connectivity between several SMN and DMN ROI pairs was associated with better gait speed, Timed‐Up‐and‐Go score, and monofilament perception. The majority of ROI pairs showing statistically significant relationship with clinical measures were interhemispheric, non‐homologous, and cortical‐to‐subcortical. Elastic net analysis for graph theory metrics revealed complexity and multi‐directionality of the relationship of the individual ROIs to clinical outcomes and lesion load. There were unique sets of ROIs associated with each clinical measure for different graph metrics.

**Conclusions:**

rsFC between specific ROIs within SMN and DMN are related to lower limb performance. Non‐homologous interhemispheric ROI‐to‐ROI connectivity was featured in the analysis. Graph theory analysis demonstrates the complex role that an individual ROI has in relation to sensorimotor function of lower extremity.

**Trial Registration:**

Clinicialtrials.gov registration number NCT03666533

## Graphical abstract

Resting‐state connectivity between specific regions of interest (ROI) within SMN and DMN is related to lower limb performance. Interhemispheric connectivity of non‐homologous ROIs was associated with better function. Graph theory analysis demonstrated the complex role that an individual ROI has in relation to sensorimotor function of lower extremity.

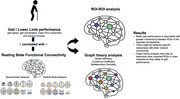

## 1 Introduction

Post‐stroke lower limb impairments and gait deficits are prevalent and lead to limitations in function and quality of life (Moore et al. 2022). Disruption of functional CNS organization is the source of these deficits (Latifi and Carmichael 2024; Baldassarre et al. 2016). Interventions directly targeting CNS organization, such as non‐invasive brain stimulation, may further enhance post‐stroke gait recovery. One barrier to development of CNS‐targeted therapies is lack of understanding of how post‐stroke functional brain organization relates to lower limb performance.

Resting‐state functional connectivity (rsFC) using magnetic resonance imaging (MRI) is well suited to study post‐stroke functional brain organization. It can be used with individuals across a broad range of impairments; simultaneous study of multiple networks can be achieved; and, importantly, connectivity can be correlated with behavioral outcomes (Carter et al. 2012). rsFC is especially useful for evaluation of gait‐related brain function because task‐related imaging is challenging for gait assessment (Horin et al. 2021). rsFC measures the temporal correlation of spontaneous brain activity among different regions in the absence of a task (Baldassarre et al. 2016). Resting‐state networks are associated with specific functions such as sensorimotor performance (Seitzman et al. 2019). Sensorimotor network (SMN) is a logical substrate for evaluation of a motor behavior such as gait and in non‐stroke elderly population, rsFC of SMN is related to gait speed (Yuan et al. 2015). In stroke survivors, studies found alterations in rsFC of SMN compared to healthy controls (Chen et al. 2019), and changes in SMN in response to motor training have been reported, particularly in upper limb stroke studies (Carter et al. 2010; Kraeutner et al. 2021). In addition to SMN, default mode network (DMN) is relevant to a global function such as gait. DMN connectivity is heightened during a state of rest, and connectivity within DMN lessens during activity (Raichle et al. 2001). DMN is believed to be important in a number of brain functions, including self‐referential activity (Raichle 2015), and its role in disease is being heavily studied (Raichle 2015). Poorer gait performance in patients with mild cognitive impairment was related to higher connectivity within DMN (Crockett et al. 2017), and DMN connectivity is known to be altered after stroke (Chen et al. 2019; Zhang et al. 2017; Zhang 2024; Dahms et al. 2024). Recovery following stroke is associated with changes in rsFC of these networks (Wang et al. 2010; Mattos et al. 2023; Li et al. 2022; Falconer et al. 2024). However, many studies of rsFC in stroke have focused on upper limb (Wang et al. 2010; Mattos et al. 2023; Li et al. 2022) or aphasia (Falconer et al. 2024), and little is known about how rsFC of these large‐scale networks relates to lower limb performance (Perry and Peters 2021).

rsFC can be evaluated not only for a pair of regions of interest (ROIs) but also through characterization of the relationship of each ROI with all other ROIs in a network using graph theoretical analysis (Latifi and Carmichael 2024; Rubinov and Sporns 2010). Graph theory analysis is a method to characterize the functional topology of a complex network. Brain networks display small‐world organization (Fornito et al. 2016) that is seen in many complex networks and is analogous to the hubs and connections of an airline map. Small‐world organization allows for high global and local topological efficiency (Fornito et al. 2016). Graph theory analysis can explore the role of each ROI in terms of its connectivity within the whole network as well as its relationship and importance among its neighbors. For upper limb motor function after stroke, rsFC graph theory analysis has identified ROIs of global and local importance (Zhang et al. 2017; Wang et al. 2010; Yin et al. 2014; Almeida et al. 2022). It would be beneficial to also characterize functional network topology and its relationship to lower limb performance after stroke. Utilizing graph theory analysis would allow for characterization of connectivity characteristics for each node and determination of brain regions of most importance in lower limb and gait performance. Ultimately, this line of investigation could help in the identification of important targets for brain stimulation to enhance rehabilitation after stroke.

There are various options for assessing stroke‐related changes of brain networks. One option is to compare brain connectivity of stroke survivors with that of age‐matched healthy controls. Although this is a powerful method of identifying stroke‐related changes, the findings are not specific to a particular function. In other words, this method does not evaluate changes that are specifically related to motor function such as gait. Therefore, an appealing option for relating stroke‐related functional changes is to correlate brain connectivity with behavioral measures of movement performance.

Our purpose is to describe the relationship of rsFC and lower limb performance in chronic stroke survivors. For our networks, we used an a priori selection of ROIs for the cortical SMN (cortical SMN), the SMN (which included both cortical and subcortical ROIs), and the DMN. In addition to ROI‐to‐ROI functional connectivity analysis, we applied graph theory analysis to further describe network topology. Our hypothesis is that better lower limb performance is associated with specific patterns of connectivity within the SMN and DMN and that both analytical approaches can provide complementary information regarding the relationship between brain function and behavior.

## 2 Methods

### 2.1 Participants

This is a cross‐sectional analysis of baseline data of ambulatory chronic stroke survivors who participated in a gait training study. Clinical measures and MRI were performed within 1–2 weeks of each other. Key inclusion criteria were unilateral stroke ≥6 months, stance phase gait deficit, ability to undergo MRI and brain stimulation, and medically stable.

### 2.2 Assessment of Lower Limb Performance

We obtained a battery of measures assessing impairment, coordination, and function to ensure a comprehensive characterization of lower limb performance. Each measure tests somewhat different function, allowing us to create measure‐specific relationships. *Fugl‐Meyer for Lower Limb (FM)* assesses lower limb motor control. Individuals move in specific motor patterns and are scored using a 3‐point scale. Movement patterns include those within a synergy (e.g., flexor synergy with simultaneous hip, knee, and ankle flexion) and out of synergy (e.g., ankle dorsiflexion with knee extended). Movement quality and reflexes are also scored. Higher scores reflect better performance (0–34 points) (Fugl‐Meyer et al. 1975). *Ten‐meter walk test* measures fastest gait speed (fGS; m/s) (Rossier and Wade 2001) averaged across three trials. *Two‐minute walk test* measures *preferred* gait speed (pGS; m/s) (Rossier and Wade 2001). *Gait Assessment and Intervention Tool (GAIT)* measures gait coordination whereby lateral and anterior–posterior views of the impaired limb are scored, with lower score indicative of better gait coordination (Daly et al. 2022). *Timed Up and Go (TUG)* assesses rising from a chair, walking 3 m, and then returning to sit (Podsiadlo and Richardson 1991). Time was averaged across three trials. *Functional gait assessment (FGA)* assesses postural stability during 10 functional walking task items (Lin et al. 2010). Individuals perform various functional walking tasks such as walking backwards, walking with eyes closed, stepping over objects, and changing direction while walking. Performance of each task is scored using a 4‐point scale, with higher score, indicating better performance (0–30 points).

Sensory function was assessed bilaterally with proprioception test, monofilament perception test, and vibration perception threshold detection. *Proprioception test* was collected with a custom‐built device designed similar to the proprioception apparatus for upper limb testing (Carey et al. 1993). The foot is placed on a plate and passively moved into plantarflexion or dorsiflexion from a neutral starting position. The order of degrees (5, 10, or 20) and direction of the deflections were randomized, and error of detection was calculated. *Semmes‐Weinstein Monofilament testing* included multiple gauges (3.61, 4.31, 4.56, 5.07, 6.10, and 6.65 mm) and testing locations (dorsum of foot, big toe, medial arch, lateral arch, and medial side of heel) (Jeng et al. 2000). The finest gauge sensed was summed across testing locations. *Vibration perception threshold* of the lateral malleolus was determined using a Biothesiometer (Bio‐Medical Instrument Co, Newbury, OH). Vibration perception threshold (in microns) was averaged across three trials. For each sensory measure, asymmetry between limbs was computed as unaffected minus affected limb score divided by the sum of the 2 scores with negative values corresponding to greater impairment in the affected limb compared to unaffected.

### 2.3 MRI Scanning Parameters

Data were collected with two scanners, a Philips Achieva 3T and General Electric Signa. Participants kept their eyes closed during scanning. Data were acquired with an 8‐channel (Philips) and a 48‐channel (General Electric Signa) head coil. Images included high resolution T1‐weighted MP‐RAGE image (1 mm^3^ voxels), T2 FLAIR image (0.7 × 0.95 × 4 mm^3^ voxels), and 10‐min functional MRI (fMRI) scan using echo planar imaging (48 slices with no gap; 3 mm^3^ voxels; TR = 3000 ms; 200 images).

### 2.4 ROI Selection

**FIGURE 1:**
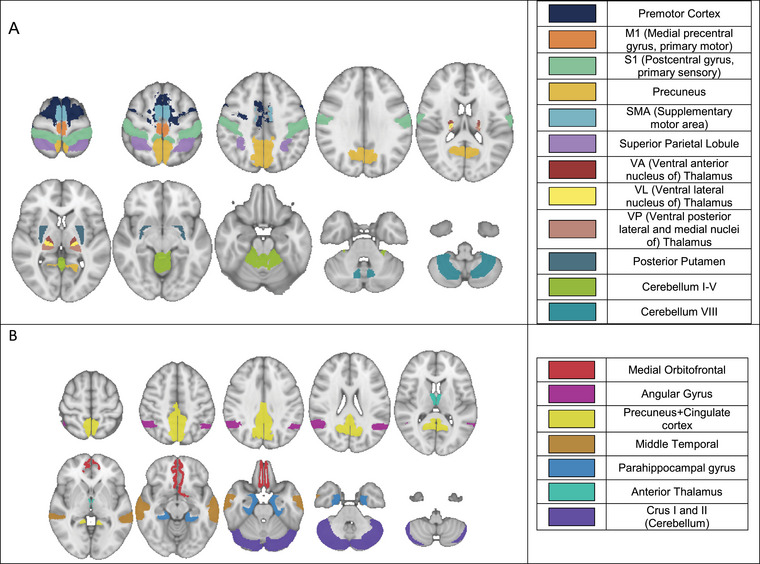
SMN ROIs (panel A) and DMN ROIs (panel B). The ROI citations are the studies supporting our choice of including the ROI in the networks.

In defining SMN, we were motivated to ensure a comprehensive representation of whole‐brain sensorimotor structures. Cortical regions associated with the SMN have been well defined, but the subcortical and cerebellar areas are not. Several functional atlases were considered (Power et al. 2011; Seitzman et al. 2020; Thomas Yeo et al. 2011; Guell and Schmahmann 2020; Gordon et al. 2016). We used Seitzman et al. (2020) atlas to define SMN ROIs, but instead of the spherical ROIs, we included the full volumes of the functional structures. This was achieved using the MNI (Montreal Neurological Institute) MRI brain template (Grabner et al. 2006) segmented using the Desikan‐Killany cortical atlas (Desikan et al. 2006), FreeSurfer thalamic parcellation (Tregidgo et al. 2023), and AAL cerebellum atlas (Rolls et al. 2020). ROIs comprising the SMN and DMN were selected based on previous publications (Guell 2020, Desikan 2006, Jones 2013, Smith 2002, Green 2015) and are shown in Figures 1A & 1B. Cortical SMN ROIs (first six ROIs in Figure 1A) were used in a separate analysis as a most commonly used SMN (Seitzman et al. 2020; Kandel et al. 2013).

### 2.5 MRI Data Pre‐Processing

fMRI scans were preprocessed in Conn‐toolbox (https://web.conn‐toolbox.org) (Whitfield‐Gabrieli and Nieto‐Castanon 2012). This included motion correction with realignment to the first scan, slice timing correction to the middle of the acquisition time, smoothing with 8 mm FWHM Gaussian kernel, outlier detection using framewise displacement of 0.9mm or global BOLD (blood oxygen level dependent) signal changes of five standard deviations, segmentation of gray and white matter, and normalization to the MNI template brain. Denoising involved temporal bandpass filtering to remove frequencies < 0.008 and > 0.9 Hz (Hallquist et al. 2013), linear regression of white matter, cerebrospinal signal and motion parameters, and scrubbing of outlier scans. Mean framewise displacement for the group was 0.3 mm, and denoising measures reduced the group variance of the voxel‐to‐voxel connectivity values from 0.4 to 0.2.

In FSL (https://fsl.fmrib.ox.ac.uk/fsl/fslwiki/) (Jenkinson et al. 2012), a lesion mask was manually created using T1 and T2 images. It was then transformed into fMRI space and used to remove the fMRI signal within the lesion area prior to data preprocessing. Scans with left hemisphere lesions were flipped left‐to‐right so that all lesions aligned to the right side.

**FIGURE 2:**
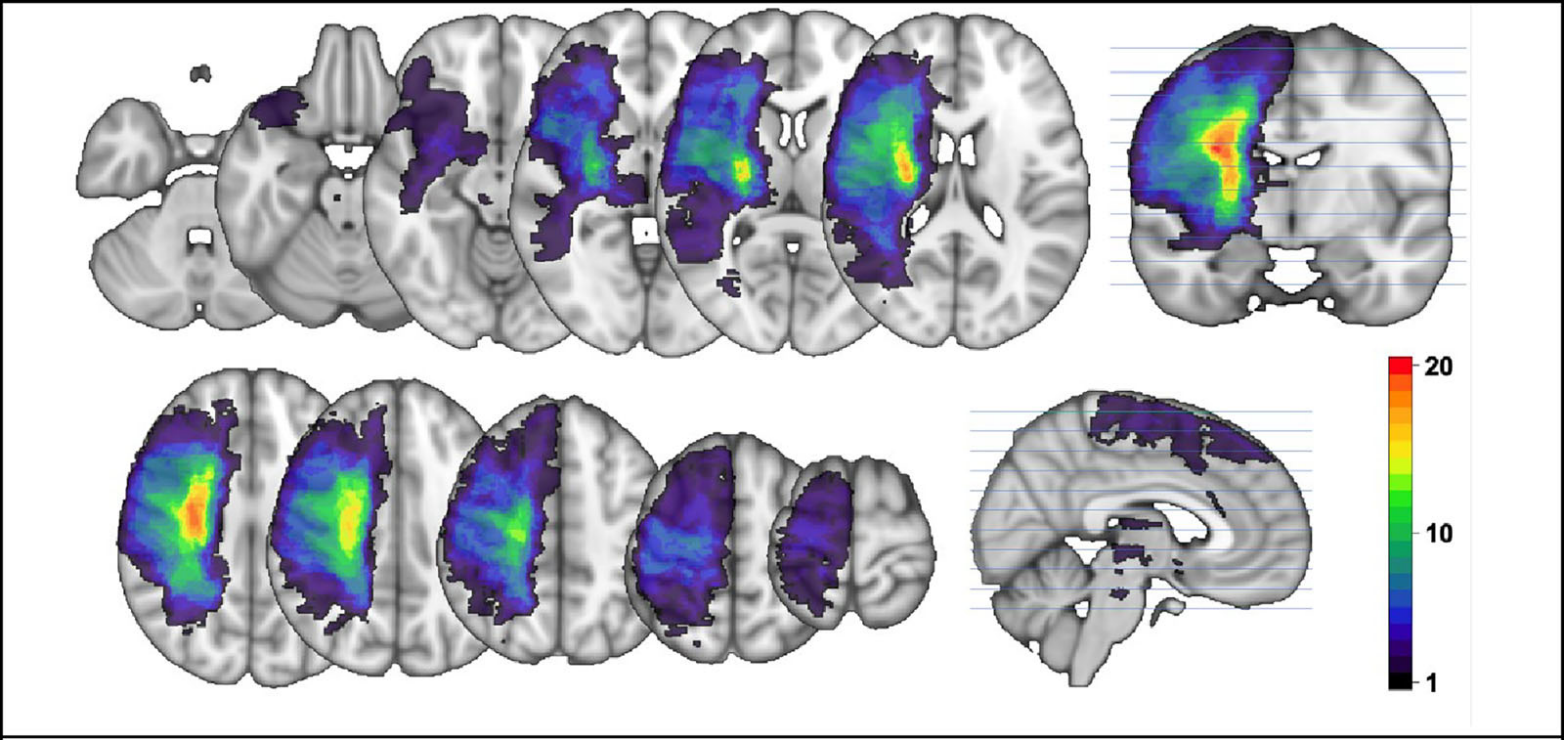
Lesion density map. Subject lesion masks are mapped onto the MNI template brain. Color bar: red = higher number of subjects with lesion in that area. Blue = lower number of subjects with lesion in that area.

Lesion load was calculated. Lesion load is the volume of lesion affecting corticospinal tract (CST) and incorporates weighting dependent on the CST level where the lesion is located (Zhu et al. 2010). The CST from the XTRACT template brain (Warrington et al. 2020), defined in MNI space, was transformed to subject space. The lesion mask (Figure 2) was overlaid with the CST, and the number of overlapping voxels in each axial slice was weighted, giving higher weight to narrower portions of the CST, and summated.

### 2.6 ROI‐to‐ROI Connectivity Analysis

ROI‐to‐ROI connectivity analysis was performed for each network using Conn‐toolbox (Whitfield‐Gabrieli and Nieto‐Castanon 2012). The Pearson correlation was computed between each ROI pair, forming a matrix of correlation coefficients that were Fisher‐transformed to increase normality. Hypothesis tests using linear regression were performed to determine associations between ROI‐to‐ROI correlation coefficients and individual clinical measures and lesion load. Correction for multiple comparisons was applied using the false discovery rate calculated using Benjamini–Hochberg method detailed in Benjamini and Hochberg (1995) and setting statistical significance at *p* < 0.05.

### 2.7 Graph Theory Metrics

The ROI‐to‐ROI correlation matrix forms the basis of a graph. A threshold is applied to the correlation matrix, forming a binary adjacency matrix (A) where above‐threshold values define the connections between ROIs, forming the graph. Threshold choice affects the number of connections in the graph and the computed graph metrics (Achard and Bullmore 2007). To avoid threshold choice bias, we computed adjacency matrices and graph metrics over a range of thresholds (0.15–0.5 at increments of 0.01) (Achard and Bullmore 2007) and calculated area under the curve (AUC) for graph metric versus threshold value plots. Other thresholds were explored. Using the methods detailed in Yin et al. (2014), we calculated the upper and lower bounds from the data; this returned upper and lower thresholds of 0.03–0.5. On examining the data, thresholds lower than 0.15 proved to be too stringent to provide reliable information as less than half the subjects had non‐zero values at a threshold below 0.15. Hence, the lower threshold bound was chosen to be 0.15 (Figures S1–S3). The final graph metrics were the AUC values that were summarized scalars independent of individual thresholds (Zhang et al. 2017; Han et al. 2020; Li et al. 2021).

Graph metrics characterize the whole graph and an ROI's relationship to other ROIs in the graph (Fornito et al. 2016). Investigating the relationship of an ROI within a network allows a deeper understanding of how important that area is for the transference of information around the network. We examined three graph theory metrics for each ROI: global efficiency (GE), betweenness centrality (BC), and clustering coefficient (CC). GE is a measure of integration and characterizes efficiency of information exchange via shortest paths (Fornito et al. 2016). GE is based on *path length*, which is the smallest number of ROIs traversed to move between two ROIs using the connections determined by the adjacency matrix. GE of an ROI is the average of the *inverse* of the path length from that ROI to all other ROIs (Rubinov and Sporns 2010). The inverse is used so that higher efficiency corresponds to shorter paths. BC is based on path lengths between all the connected ROIs. An ROI's BC is the proportion of all these paths in which the ROI appears. BC can help identify ROIs that serve as important hubs and are critical to information flow (Wang et al. 2010; Rubinov and Sporns 2010). CC reflects localized connectivity around a given ROI (Rubinov and Sporns 2010). It is based on connectivity of an ROI's neighbors. Neighbors are ROIs directly connected to a given ROI. From all possible connections between neighbors, CC is the proportion of connections that are present. Graph theory metrics were calculated in Conn‐toolbox and statistically analyzed in R.

### 2.8 Statistical Analyses Within R

Shapiro–Wilks test of normality was performed for each clinical measure. Non‐normally distributed data (lesion load and TUG) were Box‐Cox transformed (Sakia 1992). Pearson correlation was applied for clinical measures. AUC values for the graph theory metrics (GE, BC, and CC) were used for the elastic net regression analysis (Bowman et al. 2016; Tang et al. 2024).

Elastic net regression models (Zou and Hastie 2005) were used to determine if AUC graph theory metrics for each ROI were statistically associated with each clinical measure. GE, BC, and CC AUC values for each ROI were used as independent variables, and clinical measures or lesion load were the dependent variables. Separate models were used for each clinical measure and lesion load. For clinical measures, we used only complete datasets without missing values. Four clinical variables had five (monofilament, proprioception, and pGS) or six (vibration) missing observations.

For elastic net regression, fivefold cross validation was used with tuning parameter 0.05 < *α* < 1 and shrinkage penalty 0.0002 < *λ* < 0.36. One hundred tuning models were tested for each regression model, and the model with the lowest root mean squared error (RMSE) was chosen. To account for variability in cross validation random sampling with each iteration, adapting the method from Bowman et al. (2016), we repeated each of the regression models 20 times for every dependent variable. ROIs that were associated with the clinical variable at least 90% of the time were considered to have a strong association with that dependent variable and reported in the results. To help with abstraction of the results, we averaged and ranked coefficients of the significant ROIs. Following ranking, the largest coefficient was assigned 100%, and proportional values were given to the other *β* coefficients. Directionality of the relationships was on the basis of Pearson (*r*) correlation.

## 3 Results

### 3.1 Participants’ Characteristics

Participants’ characteristics are summarized in Table 1. Figure 2 shows stroke lesion location map calculated as a sum across all participants to gain the density of lesions for the cohort. The mean total lesion volume within the cohort was 49,658 mm^3^ (SD 84,882 mm^3^).

**TABLE 1:** Participants’ characteristics (*n* = 37).

Age in years, mean (SD)	64.4 (8.5)
Female (%)	16.2%
Stroke hemisphere (% Left)	56.7%
Months since stroke, mean (SD)	63.3 (54.5)
Lesion location (%)	
Cortical and subcortical	11 (30%)
Subcortical	26 (70%)
Lesion load, mean (SD)	23.7 (25.3)
FM, mean (SD)	24.1 (4.0)
fGS (m/s), mean (SD)	0.69 (0.41)
pGS (m/s), mean (SD)	0.53 (0.30)
GAIT, mean (SD)	18.5 (6.9)
TUG (s), mean (SD)	30.5 (25.2)
FGA, mean (SD)	12.5 (4.3)
Proprioception, mean (SD)	0.03 (0.46)
Monofilament, mean (SD)	−0.05 (0.10)
Vibration, mean (SD)	−0.11 (0.39)

Abbreviations: FGA, functional gait assessment; fGS, fastest gait speed; FM, Fugl‐Meyer lower limb; GAIT, Gait Assessment and Intervention Tool; pGS, preferred gait speed; TUG, Timed Up and Go.

### 3.2 Correlation of SMN ROI‐to‐ROI Connectivity With Lower Limb Performance Measures

For cortical SMN (cortical ROIs only), better GAIT and TUG scores correlated with greater connectivity between several ROI pairs (Table 3). For example, better GAIT score correlated with greater connectivity between ipsilesional (Ip) supplementary motor area (SMA) and contralesional (C) M1. Because some clinical measures have different directions of better (vs. worse) motor function, for ease of comparison, we adjusted the display of *t*‐scores in Table 3 so that the positive *t*‐score indicates that better motor function is related to higher connectivity between two ROIs. For SMN with both cortical and subcortical ROIs, there was a greater number of ROI‐pair correlations where better GAIT and TUG scores were associated with greater connectivity between those ROIs. With the exception of one ROI pair, statistically significant associations were for those that included one cortical and one subcortical ROI, and most of them were of opposite hemispheres (Table 3). For the sensory measures, better monofilament score was associated with greater connectivity between thalamic ROIs of the opposite hemisphere (Table 3).

### 3.3 Correlation of DMN ROI‐to‐ROI Connectivity With Clinical Measures

For DMN, better GAIT was associated with less connectivity between ipsilesional (Ip) precuneus + posterior cingulate and Ip anterior thalamus. Faster pGS and better TUG were associated with better connectivity between contralesional (C) and Ip precuneus + posterior cingulate (Table 3).

### 3.4 Association of Graph Theory Metrics With Lower Limb Performance and With Lesion Load

The objective of using graph theoretical approach for analysis of brain networks was to determine the relative influence of each ROI on the clinical measures. For the elastic net analysis, we used graphs for the SMN with cortical and subcortical ROIs (referred to as SMN through the rest of the manuscript) and the DMN. Table 4 includes the abstracted results of the elastic net regressions identifying ROIs with the strongest association of their graph metrics (GE, CC, and BC) with clinical measures. The negative sign in parentheses signifies that better clinical score is associated with lower graph metric value. More ROIs were identified within the SMN compared to DMN. Each clinical measure was associated with a unique set of ROIs, and the relationship varied in directionality. Here, we discuss the strongest associations.

### 3.5 Global Efficiency

For SMN, the strongest association was for better motor scores and lower GE in C premotor (for gait speed), C superior parietal (for FM), and C cerebellum I–V (for GAIT) ROIs (Table 4). Better sensory scores were associated with greater GE in Ip VP thalamus (for monofilament) and C VA thalamus (for proprioception). For DMN, better motor scores were associated with greater GE in Ip precuneus + cingulate but lower GE in Ip anterior thalamus (Table 4).

### 3.6 Clustering Coefficient

For SMN, greater CC in C VA thalamus was associated with better motor and sensory measures. However, lower CC in Ip S1 and C cerebellum was related to better motor scores (FM and GAIT, respectively), and lower CC in Ip VA thalamus and precuneus were associated with better vibration and proprioception, respectively. For DMN, lower CC in C anterior thalamus was associated with better motor and sensory scores (GAIT, monofilament, and vibration), and higher CC in Ip crus I and II was associated with better FM (Table 4).

### 3.7 Betweenness Centrality

For SMN, better clinical scores were associated with lower BC in Ip M1 (GAIT), C superior parietal (FM, monofilament), and C premotor (gait speeds) (Table 4). Higher BC in Ip VL thalamus was related to better proprioception. For DMN, lower BC in C middle temporal cortex was associated with better fGS and vibration (Table 4).

### 3.8 Lesion Load and Graph Theory Metrics

To probe the connection between the extent of structural damage to CST and residual network function, we evaluated the relationship between CST lesion load and brain connectivity. This was done to complement the inquiry of the relationship between sensorimotor deficits and functional brain connectivity. For SMN, greater lesion load was associated with higher GE in several bilateral ROIs (largest in Ip M1) and higher CC in one ROI (C cerebellum I–V) (Table 4). For DMN, greater lesion load was related to lower CC in C middle temporal gyrus and lower BC in Ip anterior thalamus (Table 4).

### 3.9 Correlation Between Lower Limb Performance Measures

To help us interpret the different relationships between brain network connectivity and various clinical measures, we evaluated correlation among the clinical measures. Strong correlations were found between the measures of gait function such as fGS, pGS, TUG, and FGA. However, functional measures (GS, TUG, and FGA) had only moderate correlation with measures of impairment and coordination (FM and GAIT). Sensory measures did not correlate with motor measures and had a moderate correlation among themselves. Correlations among the lower limb performance measures as well as lesion load are provided in Table 2.

**TABLE 2:** Correlations between clinical measures.

	fGS	pGS	GAIT	TUG	FGA	Prop	Mono	Vibr	LL
FM	0.35*	0.4*	−0.39*	−0.31	0.43**	0.21	0.17	0.50*	−0.6***
fGS		0.97***	−0.37*	−0.93***	0.75***	0.28	0.07	0.03	0.07
pGS			−0.46**	−0.93***	0.85***	0.34	0.14	0.00	0.04
GAIT				0.33*	−0.35*	−0.13	−0.14	−0.23	0.18
TUG					−0.72***	−0.32	−0.16	−0.07	−0.15
FGA						0.35	0.08	−0.10	0.03
Prop							0.59***	0.17	−0.01
Mono								0.42*	−0.27
Vibr									−0.36*

Abbreviations: FGA, functional gait assessment; fGS, fastest gait speed; FM, Fugl‐Meyer; GAIT, Gait Assessment and Intervention Tool; LL, lesion load; Mono, monofilament; pGS, preferred gait speed; Prop, proprioception; TUG, Timed Up and Go; Vibr, vibration.

*p < 0.05.

**p < 0.01.

***p < 0.001.

**TABLE 3:** Relationship between clinical measures of lower limb performance and connectivity between regions of interest (ROI) of both sensory motor (SMN) and default mode (DMN) networks.

Network	Clinical Measure	ROI‐1	ROI‐2	FDR *p* value	*T* score
SMN (cortical ROIs)	GAIT	Ip SMA	C M1	0.034	3.180
		Ip premotor	C SMA	0.029	3.240
	TUG	Ip precuneus	C precuneus	0.012	3.550
SMN (cortical and subcortical ROIs)	GAIT	Ip SMA	C VA thalamus	0.035	3.310
		Ip premotor	C VL thalamus	0.046	2.970
		Ip premotor	C VA thalamus	0.046	2.920
		C SMA	Ip post putamen	0.044	2.860
		C SMA	Ip VA thalamus	0.044	2.760
		C SMA	C VA thalamus	0.044	2.740
		C premotor	C VA thalamus	0.017	3.700
		C premotor	Ip VA thalamus	0.041	3.100
		C premotor	Ip post putamen	0.041	2.900
	TUG	Ip precuneus	C precuneus	0.025	3.550
		C M1	Ip VL thalamus	0.045	3.340
		C S1	Ip post putamen	0.031	3.480
		C premotor	Ip VA thalamus	0.022	3.610
	Mono	C VA thalamus	Ip VP thalamus	0.003	4.360
		C VL thalamus	Ip VP thalamus	0.003	4.360
		C VP thalamus	Ip VA thalamus	0.005	4.230
		C VP thalamus	Ip VL thalamus	0.005	3.980
		C VP thalamus	Ip VP thalamus	0.010	3.550
		C VL thalamus	Ip VL thalamus	0.021	3.370
		C VL thalamus	Ip VA thalamus	0.021	3.270
DMN	GAIT	Ip precuneus + cing	Ip ant thalamus	0.023	−3.400
	pGS	Ip precuneus + cing	C precuneus + cing	0.031	3.320
	TUG	Ip precuneus + cing	C precuneus + cing	0.003	4.100

*Note*: Note that there are two versions of SMN: (1) 12 cortical ROIs and (2) 12 cortical plus 12 subcortical ROIs. Results are presented as better motor or sensory function for each clinical variable as it relates to increased (positive *t*‐score) or decreased (negative *t*‐score) connectivity between two ROIs.

Abbreviations: ant thalamus, anterior thalamus; C, contralesional; DMN, default mode network; GAIT, Gait Assessment and Intervention Tool; Ip, ipsilesional; M1, primary motor cortex; Mono, monofilament; pGS, preferred gait speed; post putamen, posterior putamen; precuneus + cing, precuneus and cingulate cortex; ROI, regions of interest; S1, primary sensory cortex; SMA, supplementary motor area; SMN, sensorimotor network; TUG, Timed Up and Go; VA thalamus, ventral anterior thalamic nucleus; VL thalamus, ventral lateral thalamic nucleus; VP thalamus, ventral posterior lateral and medial thalamic nucleus.

**TABLE 4:** Elastic net analysis results showing regions of interest with strongest association of graph metrics with clinical measures and lesion load.

			Global Efficiency										Clustering Coefficient										Betweenness Centrality									
			FM	GAIT	FGA	TUG	fGS	pGS	MF	Vibr	Propr	LL	FM	GAIT	FGA	TUG	fGS	pGS	MF	Vibr	Propr	LL	FM	GAIT	FGA	TUG	fGS	pGS	MF	Vibr	Propr	LL
SMN	Ipsilesional	**M1**							**94(‐)**			**100**												**100(‐)**							**18(‐)**	
		**SMA**																														
		**Premotor**							**32(‐)**									**8**														
		**S1**											**100(‐)**							**58(‐)**	**64(‐)**										**30(‐)**	
		**Superior parietal lobule**										**52**								**26(‐)**			**37(‐)**									
		**Precuneus**							**12**			**35**									**100(‐)**		**34**								**49(‐)**	
		**VL thalamus**																													**100**	
		**VA thalamus**														**16**		**37**		**100(‐)**												
		**VP thalamus**							**100**																						**22**	
		**Post putamen**																					**28**									
		**Cerebellum I‐V**							**8(‐)**																							
		**Cerebellum VIII**						**23(‐)**																							**17(‐)**	
	Contralesional	**M1**																														
		**SMA**							**16**			**75**																				
		**Premotor**					**100(‐)**	**100(‐)**				**24**	**30**														**100(‐)**	**100(‐)**				
		**S1**																	**86(‐)**												**9(‐)**	
		**Superior parietal lobule**	**100(‐)**						**48(‐)**			**11**											**100(‐)**						**100(‐)**		**62(‐)**	
		**Precuneus**																		**51(‐)**											**34(‐)**	
		**VL thalamus**														**100**	**100**	**100**					**54(‐)**									
		**VA thalamus**							**25**		**100**								**100**	**77(‐)**			**40**								**7**	
		**VP thalamus**											**35**		**100**	**51**	**26**															
		**Post putamen**							**21(‐)**			**71**											**29(‐)**									
		**Cerebellum I‐V**		**100(‐)**			**24(‐)**	**41(‐)**			**66(‐)**		**24(‐)**	**100(‐)**								**100**	**41**									
		**Cerebellum VIII**																														
DMN	Ipsilesional	**Medial orbitofrontal**																														
		**Angular gyrus**																														
		**Precuneus+cingulate**			**100**			**100**																			**48**					
		**Middle temporal**																														
		**Parahippocampal gyrus**									**100(‐)**																					
		**Ant thalamus**				**100(‐)**	**100(‐)**	**31(‐)**																								
		**Crus**											**100**																			**100(‐)**
	Contralesional	**Medial orbitofrontal**																														
		**Angular gyrus**																														
		**Precuneus+cingulate**																														
		**Middle temporal**						**28(‐)**														**100(‐)**					**100(‐)**				**100(‐)**	
		**Parahippocampal gyrus**																														
		**Ant thalamus**												**100(‐)**					**100 (‐)**	**58(‐)**	**100(‐)**											
		**Crus**																		**100**												

*Note*: Highest model coefficient is assigned 100. Other coefficients are adjusted proportionally. Correlations are positive (better clinical measure, higher graph metric) unless marked with (−) indicating negative correlation.

Abbreviations: ant thalamus, anterior thalamus; DMN, default mode network; GAIT, Gait Assessment and Intervention Tool; M1, primary motor cortex; Mono, monofilament; pGS, preferred gait speed; post putamen, posterior putamen; precuneus + cing, precuneus and cingulate cortex; S1, primary sensory cortex; SMA, supplementary motor area; SMN, sensorimotor network; TUG, Timed Up and Go; VA thalamus, ventral anterior thalamic nucleus; VL thalamus, ventral lateral thalamic nucleus; VP thalamus, ventral posterior lateral and medial thalamic nucleus.

## 4 Discussion

We found a relationship between rsFC in large‐scale brain networks and lower limb performance in chronic stroke survivors using both ROI‐to‐ROI connectivity analysis and graph theory analysis. Addition of subcortical regions into SMN provided more detail about the relationship of clinical outcomes and rsFC. This extended SMN showed associations between clinical measures and connectivity with thalamus/putamen in addition to cortical SMN regions. By applying graph theory, we assessed the role of each ROI relative to the whole network and among its close neighbors for relationship with individual sensorimotor functions.

Greater rsFC between SMN ROI pairs was associated with better clinical scores. Better motor performance was associated with greater connectivity between cortico‐cortical pairs, for example, SMA/premotor/M1/S1. By including subcortical ROIs into SMN, we found better motor performance was also related to cortico‐deep gray matter structure connectivity (e.g., thalamus and putamen). Expansion of SMN with subcortical ROIs led to discovery of functionally important interhemispheric cortico‐subcortical connectivity as it relates to lower limb clinical performance.

The relationship between rsFC and lower limb performance after stroke has not yet been well explored. There are a few reports with mixed findings and using variable methodologies (Cui et al. 2022; Park et al. 2011; Collett et al. 2021). Similarly to our findings, those studies suggest that ROI‐to‐ROI connectivity is associated with motor function (Cui et al. 2022; Park et al. 2011; Collett et al. 2021). In an acute stroke treatment study, mirror therapy combined with conventional lower limb rehabilitation resulted in increased functional connectivity between ipsilesional M1 and superior frontal gyrus, thalamus, paracentral lobule, and postcentral gyrus (Cui et al. 2022). These changes were found in parallel with greater improvements on lower limb clinical assessments such as FM and Berg Balance Scale (Cui et al. 2022). In a longitudinal observational study early after stroke, rsFC between ipsilesional M1 and contralesional thalamus, SMA, and middle frontal gyrus correlated with improvement on overall FM score (including both upper and lower scales) at 6 months (Park et al. 2011). In a chronic stroke study, the rsFC of contralesional M1 at baseline was greater with ipsilesional precentral gyrus, superior frontal gyrus, and SMA in a poorer walking group compared to better walking group, whereas baseline rsFC of ipsilesional M1 was not different between the groups (Collett et al. 2021). Although in these studies the network building was restricted to connectivity with the M1 region (Cui et al. 2022; Park et al. 2011; Collett et al. 2021) and some evaluated lower limb function longitudinally over the early recovery period (Cui et al. 2022; Park et al. 2011), just as in our study, they signify the importance of motor network connectivity in lower limb function. Our study using a more comprehensive network analysis demonstrates a wider range of ROI pairs where greater connectivity is related with better scores on functional lower extremity measures (TUG and GAIT).

With striking consistency, our results point to the importance of interhemispheric connectivity between both cortical–cortical and cortical–subcortical ROI pairs in lower limb function. Our analysis yielded only 2 ROI pairs on the same hemisphere (contralesional VA thalamus and SMA/premotor cortices in SMN), whereas there were 23 interhemispheric ROI‐pair connectivities that correlated with better function, most of which were between non‐homologous regions (e.g., Ip SMA‐C M1 or Ip premotor‐C thalamus). Of note, one intrahemispheric ROI‐pair connectivity was associated with worse motor function (ipsilesional precuneus and anterior thalamus in DMN). Other studies documented a similar favorable interhemispheric pattern observed longitudinally over the first 6 months of recovery (Park et al. 2011) and in a cross‐sectional study (Carter et al. 2010). Interhemispheric functional connectivity of ipsilesional M1 and contralesional thalamus, SMA, and middle frontal gyrus in acute stroke were positively correlated with improvement on FM at 6 months (upper and lower limb portions) (Park et al. 2011). In acute stroke, better interhemispheric connectivity in attention network was found to be significantly associated with better gait measures (Carter et al. 2010), although they were unable to identify a significant relationship for the somatomotor network ROIs (Carter et al. 2010). In this study, the authors describe superiority of interhemispheric homologous connectivity in predicting better outcomes compared to non‐homologous and intrahemispheric connectivity (Carter et al. 2010). In our study, most of the interhemispheric connectivity related to better motor scores was for non‐homologous ROIs. There are a few possible reasons for the differences. First, their SMN was derived from the upper limb task‐related fMRI activation pattern (Carter et al. 2010). Second, lower limb function might be more dependent on non‐homologous interhemispheric connectivity than upper limb. Finally, their study was based on an acute post‐stroke population, wherein the state of brain network connectivity is expectedly different from those in the chronic phase. Overall, there is an apparent importance of interhemispheric connectivity in lower limb motor function that may go beyond the homologous areas.

Ipsilesional M1 connectivity did not feature in our ROI–ROI analysis results. Instead, our findings highlight non‐homologous FC for bilateral premotor cortex, SMA, occasionally contralesional M1 and S1, and precuneus. Others have also suggested the importance of non‐primary motor regions, such as SMA in stroke motor recovery (Park et al. 2011; Mihara et al. 2021) as well as SMA/premotor area in the gait of elderly (Yuan et al. 2015). Following fNIRS‐guided neurofeedback training for gait and balance after stroke, change in rsFC between bilateral SMA and contralesional inferior frontal gyrus correlated with improvement on Berg Balance Scale (Mihara et al. 2021), suggesting the importance of SMA in balance recovery. Here, we extend the literature by suggesting additional importance of SMA/premotor in gait coordination (GAIT) and function (TUG) in chronic stroke, especially their connectivity with contralesional SMN regions.

Homologous interhemispheric rsFC of bilateral precuneus was found to be related to TUG within SMN. Moreover, for DMN, interhemispheric rsFC of precuneus + cingulate ROIs correlated with better gait speed and TUG. Similarly, others found ipsilesional precuneus FC with ipsilesional M1 to be important for good walkers (Collett et al. 2021). Right precuneus connectivity was higher in healthy versus stroke during simulated walking (Al‐Yahya et al. 2016), and in older adults, increased bilateral precuneus activity was associated with better fast walking speed and faster obstacle navigation. (Gonzales et al. 2019) Therefore, homologous precuneus connectivity seems to be important for gait speed‐related measures.

Inter‐thalamic connectivity stood out in association with sensory impairment (monofilament perception). Others have shown better sensory function has significant positive impact on lower limb motor performance for the aged population (Lipsitz et al. 2018) and for individuals with stroke (Kim et al. 2020). However, to our knowledge, no studies have assessed relationship between functional brain connectivity and light touch perception of the lower limb in chronic stroke. Studies of upper limb somatosensory testing in chronic stroke demonstrated a difference in interhemispheric connectivity compared to healthy controls (Goodin et al. 2018), and the return of interhemispheric S1 connectivity in association with improved touch sensation (Bannister et al. 2015). Our findings for the lower limb support observations in upper limb studies that interhemispheric connectivity is important for light touch perception.

Graph theoretical approach and elastic net regression analysis allowed us to further assess the relative impact of each ROI on lower limb performance. Overall, our findings point to a heterogeneous and complex nature of the relationship between brain rsFC and sensorimotor function. Each clinical measure was associated with a different set of ROIs. In our results, we chose to highlight ROIs with the highest *β* coefficients in elastic net models.

GE is a metric of global connectedness for each node in the network. We found that for SMN, better motor function was associated with lower contralesional GE in premotor, superior parietal, and cerebellum. In contrast, better sensory function was associated with both higher and lower GE for different ROIs. For DMN, better motor function was associated with higher GE in Ip precuneus + cingulate and lower GE in Ip anterior thalamus; better proprioception was related to lower GE in parahippocampal gyrus. GE in DMN was less consistent, with higher and lower values associated with different clinical measures. Although the pattern of associations is complex and multi‐directional, the tendency for lower GE to be associated with better function agrees with other studies that correlated motor function after stroke and functional small‐world organization of the brain.

In our study, lower GE was related to better function (SMN: GAIT; DMN: fGS, pGS). An upper limb study investigating functional network changes after undergoing an arm reaching training program in chronic stroke patients found reduction in GE following training (Kraeutner et al. 2021). This study has similar findings to ours that lower GE is associated with better function. Although, using a comprehensive clinical assessment battery, our study demonstrates variability in direction and location of significant associations.

CC is a measure of local connectivity of neighboring ROIs. For FGA, TUG, fGS, pGS, and monofilament, better score was associated with higher CC in C thalamus. On the other hand, for CC in Ip thalamus, better vibration and proprioception were associated with lower CC. This suggests that functional clusters within the contralesional rather than the ipsilesional hemisphere may play a role in supporting function after stroke.

To our knowledge, investigations of CC in lower limb motor performance are limited. However, similarly to our findings, a study that observed improvement in TUG and Berg Balance Scale, as a result of VR‐based training showed both increases and decreases in CC in response to therapy (Feitosa et al. 2023). The VR group performed better with therapy and showed increased CC in bilateral frontoparietal executive regions, bilateral somatomotor network, Ip motor cerebellum, and Ip frontoparietal task control region, and decreased CC in C thalamus and Ip associative cerebellum (Feitosa et al. 2023). The fact that the directionality of CC association with clinical performance varies among the network ROIs suggests different functionality of these functional brain clusters that support sensorimotor behavior.

To evaluate the importance of each ROI as functional hubs in the networks, we used BC. The hub‐like characteristics are analogous to those of an airline map. Interestingly, we found that better motor scores were associated with lower BC in Ip M1 (for GAIT), C superior parietal (for FM), C premotor (for fGS and pGS) in SMN, and C middle temporal (for fGS) in DMN. This suggests that having hubs in those ROIs is ineffective for this population. Positive associations between better function and BC were observed for Ip VL thalamus (for proprioception). To our knowledge, there have been no reports in the literature using BC as it relates to lower limb performance after chronic stroke. For upper limb, a few studies of acute (Wang et al. 2010; Almeida et al. 2022) and chronic (Yin et al. 2014) stroke found that BC of some ROIs exhibited positive and others negative correlation with motor function. The areas that had a negative association included Ip anterior inferior cerebellum (Wang et al. 2010), Ip thalamus (Wang et al. 2010), Ip middle frontal cortex (Yin et al. 2014), Ip superior parietal lobule (Yin et al. 2014), C anterior inferior cerebellum (Yin et al. 2014), and C SMA (Yin et al. 2014). Positive relationship with better function was reported for Ip M1 (Wang et al. 2010), C dentate nucleus (Wang et al. 2010), bilateral dorsolateral premotor cortex (Yin et al. 2014), and bilateral SMA (Almeida et al. 2022). Some evidence suggests that BC may be a measure that can be used to discern differences in functional brain topology within patient populations (Yin et al. 2014; Ruan et al. 2020). Although, currently, there may not be an agreement in specific regional importance of each network ROI, further exploration to identify key hubs within large‐scale brain networks may serve as potential targets for development of focused interventions such as brain stimulation.

Finally, we evaluated relationships of CST damage (lesion load) and graph metrics for SMN and DMN. Similarly to clinical measures, the relationship was complex. Greater lesion load was related to higher GE in several bilateral SMN ROIs (including Ip M1), suggesting that with greater structural damage, there might be a greater need for global connectivity, whereas small‐world efficiency is lacking. This is similar to the findings for our clinical measures where higher GE was associated with worse function. This is interesting because lesion load has only moderate correlation with FM (*r* = 0.6). Therefore, extent of CST damage provided an additional element in characterizing the brain's condition. Focal lesions are known to disrupt functional connectivity (Griffis et al. 2019; Carter et al. 2012; Gratton et al. 2012). However, our study emphasizes the importance to consider a non‐linear relationship between extent of CST lesion and resulting network connectivity and clinical performance.

### 4.1 Limitations

Our sample size is relatively small, though in line with other published studies of rsFC in stroke. We included individuals with a range of deficits, and therefore heterogeneity is present, though we believe the cohort represents the array of patients seen in clinical practice. We used an a priori approach to ROI selection, so other ROIs of importance may have been excluded, though we selected ROIs based on what is known about the studied networks. We did not explore how the different networks, DMN and SMN, interact with each other, and we limited our analysis to only these two networks. Future study that includes additional resting‐state networks may provide greater understanding of the hierarchical relationship of resting‐state networks as they relate to post‐stroke gait performance.

## 5 Conclusions

A whole‐brain network approach provided greater insight into how large‐scale resting‐state connectivity might be related to clinical lower limb performance. Non‐homologous interhemispheric connectivity was featured prevalently in our ROI‐to‐ROI analysis and was related to multiple clinical measures of lower limb performance. It is worthwhile to expand the analysis of connectivity to include deep brain structures (thalamus, basal ganglia, and cerebellum). Graph theory analysis provides an additional insight into complexity of motor control of gait and lower limbs. A multimodal approach that includes a battery of clinical measures and different analytical methods will be needed to fully understand how brain connectivity is related with lower limb performance after stroke.

## Author Contributions

**Svetlana Pundik**: conceptualization, data curation, formal analysis, funding acquisition, investigation, methodology, project administration, resources, supervision, visualization, writing–original draft, writing–review and editing. **Margaret M. Skelly**: conceptualization, data curation, formal analysis, funding acquisition, investigation, project administration, writing–original draft, writing–review and editing. **Jessica P. McCabe**: conceptualization, data curation, formal analysis, funding acquisition, investigation, methodology, project administration, writing–original draft, writing–review and editing. **Ahlam I. Salameh**: data curation, formal analysis, investigation, writing–original draft, writing–review and editing. **Trenley Anderson**: data curation, investigation, writing–original draft. **Kelsey Rose Duncan**: data curation, formal analysis, methodology, writing–review and editing. **Terri Hisel**: data curation, formal analysis, methodology, writing–review and editing. **Sarah J. A. Carr**: formal analysis, investigation, methodology, software, visualization, writing–original draft, writing–review and editing.

## Disclosure

The authors disclosed receipt of the following financial support for the research, authorship, and/or publication of this article.

## Ethics Statement

This study was approved by the Institutional Review Board of the VANEOHS on July 18, 2018 (IRB 15036‐H19). This research was conducted ethically in accordance with the World Medical Association Declaration of Helsinki.

## Consent

All participants provided written informed consent prior to enrollment in the study.

## Conflicts of Interest

The authors declare no conflicts of interest.

### Peer Review

The peer review history for this article is available at https://publons.com/publon/10.1002/brb3.70519.

## Supporting information



Supporting Information

## Data Availability

The data that support the findings of this study are available from the corresponding author upon reasonable request.
