# Supplementary material for: Resting‐State Functional Connectivity of Sensorimotor and Default Mode Networks and Lower Limb Performance in Chronic Stroke: A Cross‐Sectional Study
Source: Brain Behav. 2025 May 7;15(5):e70519. doi: 10.1002/brb3.70519 (PMC12056360; doi:10.1002/brb3.70519)

Supplementary Information

We include a plot here to show how subjects contributed to the three graph metrics - GE, BC and CC – at each cost function. The network consisted of 24 ROIs and analysis included 37 subjects. At each cost function and for each graph metric we counted the number of ROIs with non-zero value for each subject. In the graphs below we plot the average number of ROIs with non-zero value at each cost over all subjects. For example, for betweenness centrality at cost threshold 0.05 on average, there were 5 of 24 ROIs with non-zero values across the network. Choosing the lower boundary threshold for cost of 0.15 appeared reasonable as we were interested in ROI level information.

Figure 1:


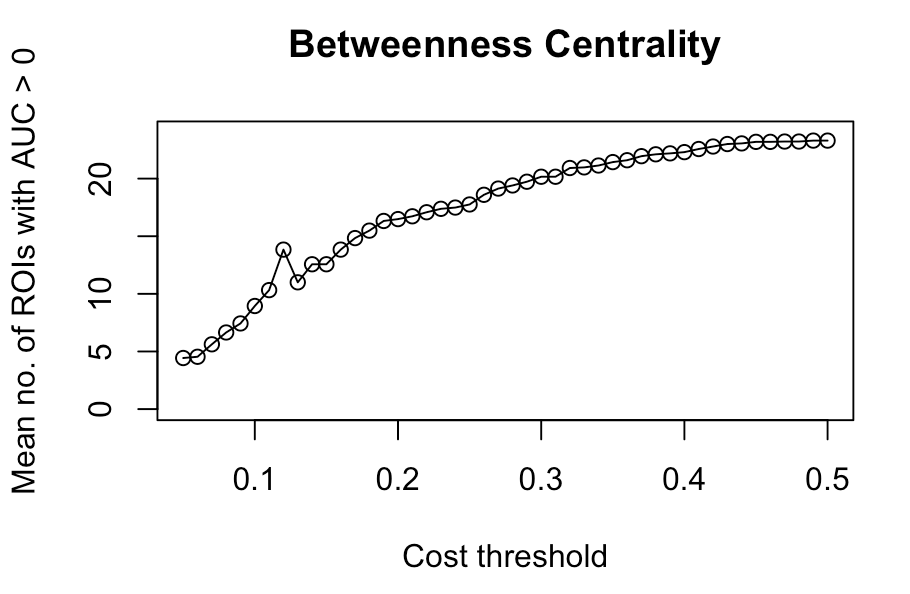


Figure 2:


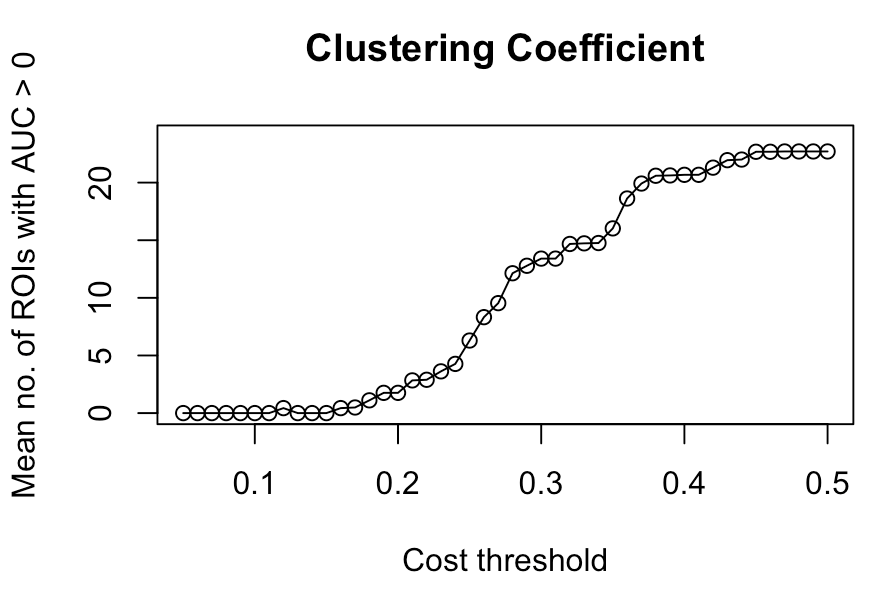


Figure 3:


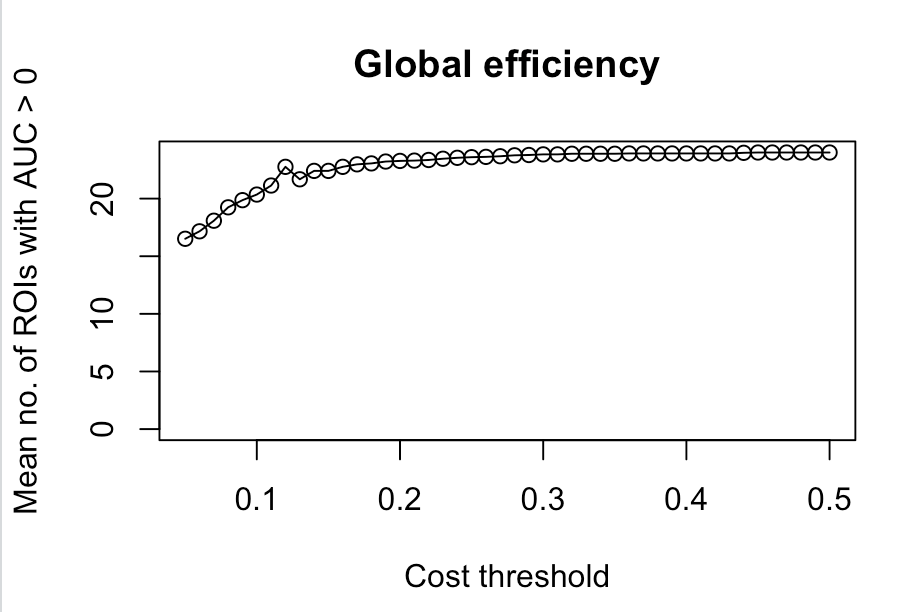

Supplement: Supplementary file 1 — Supporting Information [file BRB3-15-e70519-s001.docx]
